# Supplementary material for: Fine Particulate Matter-Induced Oxidative Stress Mediated by UVA-Visible Light Leads to Keratinocyte Damage
Source: Int J Mol Sci. 2021 Sep 30;22(19):10645. doi: 10.3390/ijms221910645 (PMC8509012; doi:10.3390/ijms221910645)
Supplement: Supplementary file 1 [file ijms-22-10645-s001.zip › ijms-1383053-supplementary.pdf]

International Journal of *Molecular Sciences*

MDPI

Fine Particulate Matter-Induced Oxidative Stress Mediated by UVA-Visible Light Leads to Keratinocyte Damage

Krystian Mokrzyński [1,†], Olga Krzysztyńska-Kuleta [1,†], Marcin Zawrotniak [2],Michał Sarna [1] and Tadeusz Sarna [1,*]

[1] Department of Biophysics, Faculty of Biochemistry, Biophysics and Biotechnology, Jagiellonian University, 30-387 Cracow, Poland; mokrzynskikrystian@gmail.com (K.M.); olga.krzysztynska@doctoral.uj.edu.pl (O.K.-K.); michal.sarna@uj.edu.pl (M.S.)

[2] Department of Comparative Biochemistry and Bioanalytics, Faculty of Biochemistry, Biophysics and Biotechnology, Jagiellonian University, 30-387 Cracow, Poland; marcin.zawrotniak@uj.edu.pl

* Correspondence: tadeusz.sarna@uj.edu.pl

† Contributed equally

Supplementary materials

Table S1. Identification of free radicals trapped using DMPO in 70%/30% DMSO/H2O.

Season	Excitation Wave-length [nm]	A_N [mT]	$A_{H\alpha}$ [mT]	$A_{H\beta}$ [mT]	Radical	Reference	R^2 of the Fit
Winter	365	1.332	1.062	0.133	Superoxide anion	[31–33]	0.96
		1.435	1.247	-	Nitrogen-centered radical		
		1.705	1.313	-	Unidentified 1		
	400	1.326	1.056	0.137	Superoxide anion		0.97
		1.426	1.256	-	Nitrogen-centered radical		
		1.716	1.295	-	Unidentified 1		
	440	1.341	1.070	0.133	Superoxide anion		0.92
		1.439	1.278	-	Nitrogen-centered radical		
		1.722	1.323	-	Unidentified 1		
	540	1.330	1.058	0.127	Superoxide anion		0.60
Spring	365	1.338	1.060	0.140	Superoxide anion	[31,32,34]	0.95
		1.514	1.699	-	Sulfur-centered radical		
		1.328	1.516	-	Unidentified 2		
	400	1.333	1.065	0.140	Superoxide anion		0.95
		1.509	1.703	-	Sulfur-centered radical		
		1.330	1.498	-	Unidentified 2		
	440	1.329	1.067	0.135	Superoxide anion		0.80
		1.516	1.700	-	Sulfur-centered radical		
		1.301	1.490	-	Unidentified 2		
	540	1.322	1.072	0.132	Superoxide anion		0.50
Summer	365	1.338	1.059	0.141	Superoxide anion	[31,32,34]	0.97
		1.508	1.701	-	Sulfur-centered radical		
	400	1.335	1.065	0.140	Superoxide anion		0.96
		1.516	1.707	-	Sulfur-centered radical		
	440	1.338	1.068	0.135	Superoxide anion		0.75
		1.517	1.694	-	Sulfur-centered radical		
	540	1.336	1.066	0.133	Superoxide anion		0.55
Autumn	365	1.326	1.060	0.128	Superoxide anion	[31–33]	0.96
		1.426	1.242	-	Nitrogen-centered radical		
		1.700	1.346	-	Unidentified 1		
	400	1.327	1.056	0.133	Superoxide anion		0.97
		1.421	1.248	-	Nitrogen-centered radical		
		1.699	1.341	-	Unidentified 1		
	440	1.323	1.052	0.132	Superoxide anion		0.95
		1.423	1.265	-	Nitrogen-centered radical		
	540	1.311	1.049	0.125	Superoxide anion		0.50

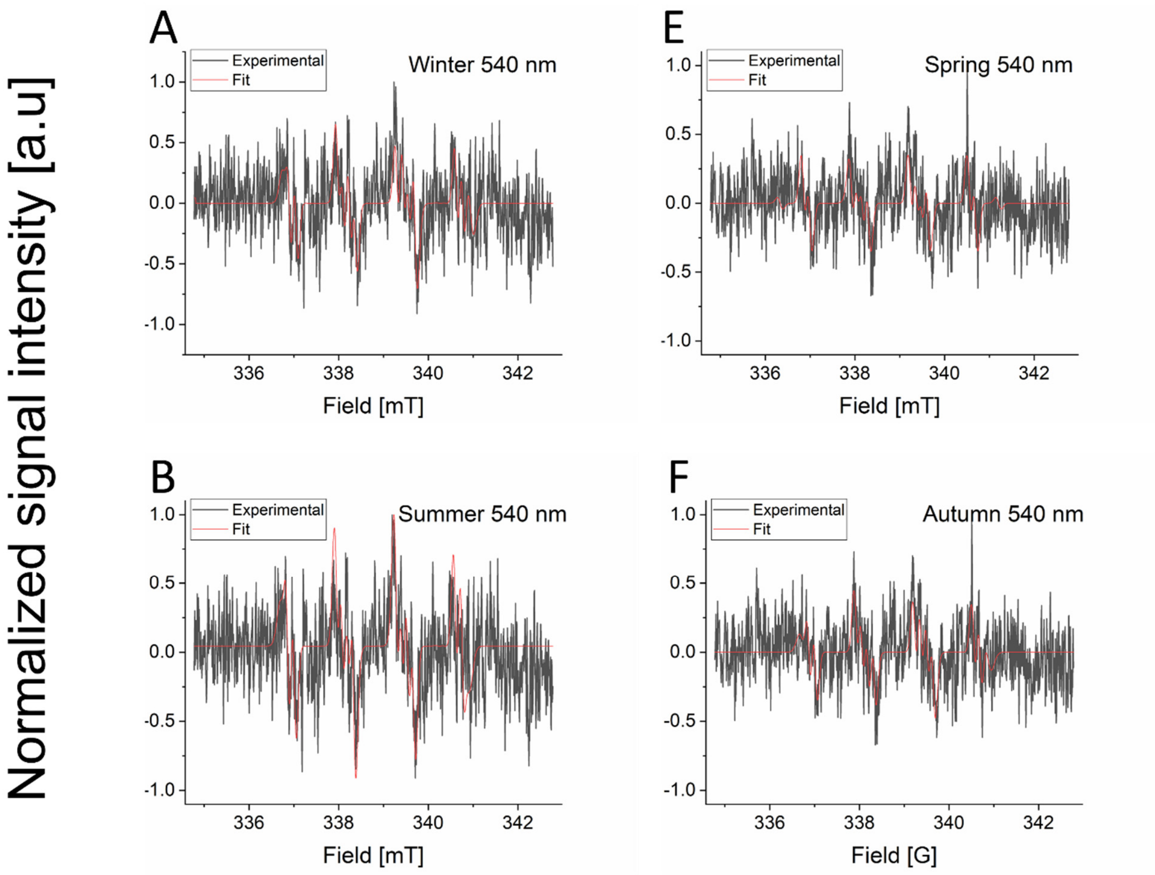

Figure S1. EPR spin-trapping of free radicals generated by $PM_{2.5}$ samples from different seasons: Winter (**A**), Spring (**B**), Summer (C) and Autumn (**D**) during 540 nm irradiation. Black lines represent spectra of photogenerated free radicals trapped with DMPO, red lines represent the fit obtained for the corresponding spectra.

Table S2. Initial velocities [a.u/min] of photoproduction of free radicals adducts with DMPO spin trap.

Excitation Wavelength [nm]	Winter			Spring			Summer		Autumn		
	**Superoxide anion**	***N*-centered radical**	**Unidentified radical 1**	**Superoxide anion**	***S*-centered radical**	**Unidentified radical 2**	**Superoxide anion**	***S*-centered radical**	**Superoxide radical**	***N*-centered radical**	**Unidentified radical 1**
365	$9.07 \times 10^{-2} \pm 6.35 \times 10^{-3}$	$2.73 \times 10^{-2} \pm 1.64 \times 10^{-3}$	$3.63 \times 10^{-2} \pm 2.54 \times 10^{-3}$	$2.73 \times 10^{-2} \pm 2.52 \times 10^{-3}$	$5.00 \times 10^{-2} \pm 4.50 \times 10^{-3}$	$6.39 \times 10^{-3} \pm 5.75 \times 10^{-4}$	$7.77 \times 10^{-2} \pm 7.42 \times 10^{-3}$	$4.39 \times 10^{-2} \pm 3.08 \times 10^{-3}$	$4.67 \times 10^{-2} \pm 1.87 \times 10^{-3}$	$1.97 \times 10^{-2} \pm 1.58 \times 10^{-3}$	$2.80 \times 10^{-3} \pm 1.68 \times 10^{-4}$
400	$3.03 \times 10^{-2} \pm 1.21 \times 10^{-3}$	$1.04 \times 10^{-2} \pm 8.28 \times 10^{-4}$	$8.85 \times 10^{-3} \pm 4.42 \times 10^{-4}$	$2.36 \times 10^{-2} \pm 4.71 \times 10^{-4}$	$3.14 \times 10^{-2} \pm 6.28 \times 10^{-4}$	$6.14 \times 10^{-4} \pm 3.07 \times 10^{-5}$	$2.64 \times 10^{-2} \pm 5.28 \times 10^{-4}$	$1.58 \times 10^{-2} \pm 9.48 \times 10^{-4}$	$1.91 \times 10^{-2} \pm 2.11 \times 10^{-3}$	$1.12 \times 10^{-2} \pm 7.82 \times 10^{-4}$	$5.43 \times 10^{-3} \pm 3.80 \times 10^{-4}$
440	$1.09 \times 10^{-2} \pm 9.77 \times 10^{-4}$	$3.86 \times 10^{-3} \pm 1.16 \times 10^{-4}$	$1.01 \times 10^{-3} \pm 7.06 \times 10^{-5}$	$1.88 \times 10^{-3} \pm 1.50 \times 10^{-4}$	$3.32 \times 10^{-4} \pm 1.99 \times 10^{-5}$	$7.60 \times 10^{-4} \pm 4.54 \times 10^{-5}$	$4.96 \times 10^{-3} \pm 2.97 \times 10^{-4}$	$2.62 \times 10^{-4} \pm 1.83 \times 10^{-5}$	$7.85 \times 10^{-3} \pm 1.57 \times 10^{-4}$	$3.29 \times 10^{-3} \pm 2.63 \times 10^{-4}$	-

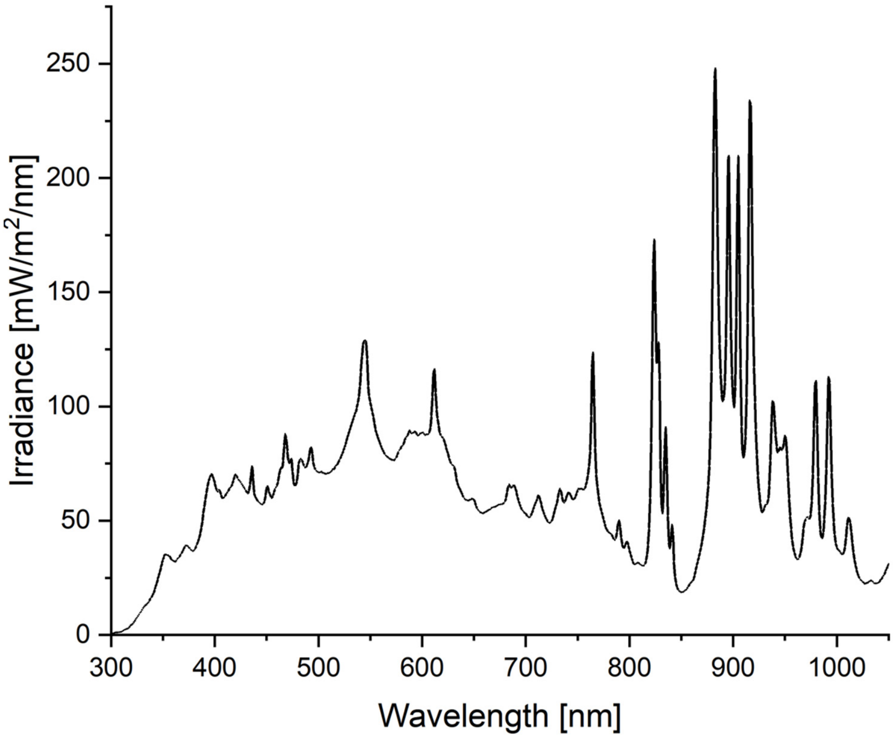

Figure S2. Irradiance of solar simulator ScienceTech SS1.6 kW equipped with AirMass 0 filter and 330 nm cut-off filter.
